# Functional properties of beetroot (*Beta vulgaris*) in management of cardio-metabolic diseases

**DOI:** 10.1186/s12986-019-0421-0

**Published:** 2020-01-07

**Authors:** Parvin Mirmiran, Zeinab Houshialsadat, Zahra Gaeini, Zahra Bahadoran, Fereidoun Azizi

**Affiliations:** 1 Nutrition and Endocrine Research Center, Research Institute for Endocrine Sciences, Shahid Beheshti University of Medical Sciences, Tehran, No. 24, Sahid-Erabi St, Yemen St, Chamran Exp, Tehran, Iran; 2 Endocrine Research Center, Research Institute for Endocrine Sciences, Shahid Beheshti University of Medical Sciences, Tehran, Iran

**Keywords:** Beetroot, Hypertension, Diabetes, Kidney function, Nitric oxide

## Abstract

Red beetroot (*Beta vulgaris*), as a naturally occurring root vegetable and a rich source of phytochemicals and bioactive compounds, is known for its beneficial roles in the improvement of several clinical and pathologic outcome. Chronic and acute beetroot juice supplementation, as a cost-effective strategy, is proposed to hold promises in controlling diabetes and insulin hemostasis, blood pressure and vascular function, renal health and the possible effect on microbiome abundance. The secondary outcome and physiological response of microbiome abundance modulation included the non- significant fluctuation of systolic and diastolic blood pressures. Also, some studies have suggested a reno-protective property of beetroot juice that is associated with the reduction of mortality rate and favorable changes in kidney’s functional parameters among patients with renal disorders. Similarly, it is shown that the persistent consumption of beetroot juice effectively postpones the postprandial glycemic response and decreases the blood glucose peak. The significant blood pressure lowering effect has been seen among normotensive subjects, which tend to be more considerable among hypertensive individuals and progressive among overweight adults.

Within this context, this review aims to provide a comprehensive overview on the therapeutic applications of beetroot juice in metabolic disorders and theirs underlying mechanisms. Despite the inconsistencies in the set of results from the reviewed studies, there is no doubt that further contributing factors must be investigated more deeply in future studies.

## Introduction

Beetroot, an annual or biennial cultivated form of *Beta vulgaris subsp. vulgaris conditiva*, includes a variety of edible taproots originated from the Middle East, which has been spreading worldwide, from the Americas to Europe and Asia [1, 2]. As a rich and nutritious source, it is believed to hold health-promotional characteristics, anti-oxidant and anti-inflammatory effects [3], anti-carcinogenic and anti-diabetic activities and hepato-protective, hypotensive and wound healing properties [4, 5]. Therefore, beetroot is currently being applied as a functional ingredient in the development of various meals [6, 7]. It is notable that most recent studies on beetroot supplementation, especially those addressing its hypotensive and ergogenic properties, emphasized the critical role of inorganic NO_3_ on the clinical effect of this vegetable and its byproducts.

So far, various interventional studies from selective literature have explored and addressed the implications of beetroot and its byproducts on systolic and diastolic blood pressures, vascular and endothelial function, insulin and glucose responses within the glycemic homeostatic context, and the abundance of microbiome. The overall results were ultimately found to be mostly inconsistent. Also, the hypotensive and hypoglycemic effect of beetroot juice consumption had not been firmly attributed to one and major responsible mechanism; such analytic vision was seen across microbial and renal studies as well.

This comprehensive review provided a detailed, reliable proof on the treatment of the elevated renal parameters including renal resistive index and arterial stiffness with beetroot and its components. Additionally, within this review we aim to provide an updated summery of beetroot consumption and its ultimate effects on blood glucose, blood pressure and microbiome levels, vascular and renal function and therefore, incidence of metabolic syndrome. Findings from this review are useful in addressing mechanisms involved in key metabolic areas and a wrap up on different aspects of each study.

The achievement of this goal paves the way of taking further pharmacological and nutritional advantages in the prevention and treatment levels and bring new perspectives into such multidisciplinary field. With the constant evolving matter of science, this review is one of its kind in the past few years that reported the metabolic effect of beetroot juice on different populations.

### Nutrients and bioactive compounds of beetroot

Beetroot is consist of multiple biologically active phytochemicals including betalains [8] (e.g., betacyanins and betaxanthins), flavonoids, polyphenols, Saponins [8] and inorganic Nitrate (NO_3_); it is also a rich source of diverse minerals such as potassium, sodium, phosphorous, calcium, magnesium, copper, iron, zinc and manganese [9]. It is commonly consumed in form of supplemental juice, powder, bread, gel, boiled, oven-dried, pickled, pureed or jam-processed across different food cultures [1, 10, 11]. As shown in Table 1, 100 mL of beetroot juice is comprised of 95 Kcal energy, 22.6 g carbohydrates, 0.70 g proteins, 0.16 g total lipids, 0.91 g total dietary fiber and 12 g total sugars. As such, the micro nutritional composition of 100 mL beetroot juice is estimated as 8.8 g sucrose, 0.86 g fructose, and 2.5 g glucose [8].

**Table 1:** Nutrient composition of beetroot and its byproducts (per 100 g or L)

	Raw	Cooked, boiled	Canned	Fresh juice
Water, g	87.58	87.06	90.96	–
Energy, kcal	43	44	31	30
Protein, g	1.61	1.68	0.91	1.02
Total fats, g	0.17	0.18	0.14	0
Carbohydrate, g	9.56	9.96	7.21	6.6
Fiber, g	2.8	2	1.8	0
Sugars, g	6.76	7.96	5.51	6.6
Calcium, mg	16	16	15	0
Iron, mg	0.8	0.79	1.82	0
Magnesium, mg	23	23	17	–
Phosphorus, mg	40	38	17	–
Potassium, mg	325	305	148	–
Sodium, mg	78	77	194	93
Zinc, mg	0.35	0.35	0.21	–
Vitamin C, mg	4.9	3.6	4.1	0
Thiamin, mg	0.031	0.027	0.01	–
Riboflavin, mg	0.04	0.04	0.04	–
Niacin, mg	0.334	0.331	0.157	–
Folate, μg	109	80	30	–
Total phenolic content^a^	255	238	192	225
Total flavonoid content^b^	260	261	173	126

^a^ As mg gallic acid equivalent (GAE)/ 100 g; ^b^ as mg rutin equivalent (RE)/100 g sample

Moreover, various commercial organic and conventional beetroot juices, are reported to contain total sugar, vitamin C and total flavonoids within a range of 1.73–7.85 g, 10.75–20.36 mg, and 2.02–2.36 mg (per 100 g), respectively [12]. Betalains make up to ~ 70–100% of phenolic composition of beetroot, limited to 0.8–1.3 g/L of fresh beetroot juice (about 60% betacyanins and 40% betaxanthins) [1].

In fact, beetroot is classified as one of the ten plants with the highest antioxidant activity [8]. It is believed to be the main commercial source of betalains, as in concentrated forms, powder, or natural dyes in gelatins, confectionery, dairy, meat, and poultry derived products [8]. According to Baião et al. flavonoids undergo changes following vegetable processing while polyphenols remain active after in vitro digestion, yet found in the highest ratio in beetroot gel than other conformations including beetroot juice [8].

NO_3_ contributes as one of the most important inorganic compounds within beetroot, the content of which is reported to vary 10-fold between single varieties [1]. NO_3_ concentration was said to be within a range of 388 ± 19.9 to 3968 ± 252 mg/L among commercial beetroot juice and 393 ± 2.23 to 2721 ± 54.4 mg/L among commercial beetroot powders. Although nitrate is relatively inert, it is yet capable of transforming status into NO_2_ through bacterial enzymatic pathways (NO_3_ reductase), which subsequently is non- enzymatically decomposed to NO in the oral cavity. The classification of the beetroot organ in terms of NO_3_ concentration from highest to lowest is as petiole, leaf, stem, root, tuber, bulb, fruit, and seed, respectively [8].

Additionally, the oxalic acid constitution of beetroot is relatively abundant [13]; average content in raw beetroot and beetroot juice equals to 94.6–141.6 mg/100 g and 300–525 mg/L, respectively. Oxalic acid, as a metal ion chelator, promotes the formation of nephroliths, and therefore, is considered as a health concern especially in patients predisposed to the kidney disease [1, 14].

With the challenge of formulating biologically safe, NO_3−_ rich beetroot supplements, various preparation methods such as beetroot juice freeze-drying for the production of beetroot powder, have initially been introduced. Red round thin beetroot chips and pseudoplastic beetroot gels are also of the most recently invented and functional forms of beetroot supplements. Beetroot chips are known to contain the highest energy content (Kcal), carbohydrate and total sugar, the highest value of Total Antioxidant Potential (TAP) and the lowest value of Total Phenolic Content (TPC), flavonoids and Saponin level. The pseudoplastic gel, as a mean of NO_3_ administration to athletes, is believed to contain the highest protein and lowest lipid content, ranking beetroot gel not as the most commonly used but most effective formulation comparing to other byproducts [8].

Despite the industrial food exploitation of red beet, sugar beet is grown commercially for sugar production due to the high content of sucrose [1]. The processing of the sugar depends on the nitrogen availability, especially in the early stages of growth [15, 16].

### Effects of beetroot on blood pressure and vascular function

The awareness regarding the impact of acute and chronic beetroot juice consumption on blood pressure and vascular function by clinical studies is rapidly rising (Tables 2 and 3). Within this review, we investigated a total of 25 human studies. The number of studies with emphasis on the blood pressure lowering properties among normotensive and hypertensive individuals in different health states, overwhelms those contradicting this outcome. The role of nitrate- nitrite pathway and that of bioactive compounds are highlighted.

**Table 2:** Chronic effects of beetroot on blood pressure and vascular function

Author	Study population	Study Design	Sample Size	Duration (days)	Dose of beetroot and its corresponding NO_3_ content	Findings
Shepherd et al. [17]	COPD Patients	Randomized, double blind, placebo controlled	13	2.5	Consumption of 70 ml of beetroot juice (~ 430 mg NO_3_) vs. NO_3_-depleted beetroot juice twice a day	No effect on DBP or SBP
Keen et al. [18]	Healthy non-smokers	Randomized, double blind, placebo controlled	6	3	Daily consumption of 70 ml of beetroot juice (~ 450 mg NO_3_)	↓ Mean arterial BP, ↓ DBP
Kelly et al. [19]	Healthy adults	Randomized, double blind, cross-over	20	3	Daily consumption of 140 ml of beetroot juice (~ 595 mg NO_3_) vs. NO_3_-depleted beetroot juice	↓ Mean arterial BP, ↓ SBP and DBP
Bailey et al. [20]	Healthy adults	Randomized, double blind, cross-over	9	6	Daily consumption of 140 mL beetroot juice (~ 520 mg NO_3_) vs. no intervention	↓ Mean arterial BP, ↓ SBP and DBP
Bailey et al. [21]	Normotensive men	Double-blind, cross-over placebo-controlled	8	6	Daily consumption of 500 mL beetroot juice (~ 690 mg NO_3_) vs. blackcurrant Juice	↓ SBP
Bailey et al. [21]	Healthy active men	Randomized, double blind, cross-over	9	6	Daily consumption of 500 mL beetroot juice (~ 320 mg NO_3_) vs. blackcurrant Juice	↓ Mean arterial BP, ↓ SBP and DBP
Cermak et al. [22]	Normotensive men	Double-blind, repeated-measures cross-over	20	6	Daily consumption of 140 ml of beetroot juice (~ 490 mg NO_3_) vs. NO_3_-depleted beetroot juice	No effect on DBP or SBP
Lansley et al. [23]	Healthy active men	Randomized, double blind, cross-over	9	6	Daily consumption of 500 ml of beetroot juice (~ 3 mg NO_3_) vs. NO_3_-depleted beetroot juice	↓ SBP, no effect on DBP or arterial BP
Bailey et al. [24]	Healthy smokers and healthy non-smokers	Double-blind, cross-over	17	6	Daily consumption of 140 mL beetroot juice (~ 520 mg NO_3_) vs. NO_3_-depleted beetroot juice (~ 5 mg NO_3_)	↓ SBP in non-smokers
Lara et al. [25]	Overweight and obese adults	randomized, non-blinded Parallel design,	30	7	Daily consumption of 70 mL beetroot juice (~ 600 mg NO_3_) vs. no intervention	No effect on resting or ambulatory BP, pulse wave velocity or arterial distensibility, no change in asymmetric dimethylarginine (ADMA)
Bondonno et al. [26]	Hypertensive adults	randomized, double-blind placebo-controlled, cross-over	27	7	Daily consumption of 140 mL beetroot juice (~ 420 mg NO_3_) vs. NO_3_-depleted beetroot juice	No effect on BP
Kerley et al. [27]	Controlled and uncontrolled hypertensive patients	Uncontrolled, pilot	19	14	140 ml beetroot juice (~ 800 mg NO_3_)	↓ DBP and ↓arterial stiffness in uncontrolled patients
Gilchrist et al. [28]	Type 2 diabetic patients	Randomized double blind, placebo-controlled crossover	27	14	Daily consumption of 250 mL beetroot juice (~ 500 mg NO_3_) vs. NO_3_-depleted beetroot juice	No effect on BP or macro- or microvascular endothelial function
Asgary et al. [29]	Hypertensive un-treated adults	Randomized, single-blind, crossover	24	14	Daily consumption of 250 mL beetroot juice vs. 250 g cooked beetroot	↓ SBP and DBP, ↑ flow mediated dilation ↓ Intracellular adhesion molecule-1 and vascular cell adhesion molecule-1, ↓ E-selectin
Vanhatalo et al. [30]	Healthy subjects	Randomized, assessor-blind, crossover	8	15	Daily consumption of 500 mL beetroot juice (~ 322 mg NO_3_) vs. 500 mL low-calorie juice	↓ SBP and mean arterial blood pressure
Jajja et al. [31]	Overweight older adults	Parallel, randomized clinical	24	21	Daily consumption of 70 mL of concentrated beetroot juice (~ 300–400 mg NO_3_) vs. blackcurrant juice (~ 2.7 mg NO_3_)	No effect on resting clinic BP or 24-h ambulatory, ↓ home-monitoring daily SBP
Kapil et al. [32]	Hypertensive older adults	Randomized, phase2, double-blind, placebo- controlled study	68	28	Daily consumption of 250 mL beetroot juice (~ 450 mg NO_3_) vs. NO_3_-depleted beetroot juice	↓ BP, improve endothelial function, ↓ arterial stiffness
Velmurugan et al. [33]	Subjects with hypercholesterolemia	Randomized, double-blind, placebo-controlled parallel	69	42	Daily consumption of 250 mL beetroot juice (~ 370 mg NO_3_) vs. NO_3_-depleted beetroot juice	↓ SBP ↑ Flow mediated dilation, ↓pulse wave velocity, ↓ augmentation index ↓ Platelet-monocyte aggregates ↓ P-selectin expression

*NO* Nitric Oxide, *COPD* Chronic Obstructive Pulmonary Disease, *DBP* Diastolic Blood Pressure, *SBP* Systolic Blood Pressure

**Table 3:** Acute effects of beetroot on blood pressure and vascular function

Author	Study population	Study Design	Sample size	Dose of beetroot and its corresponding NO_3_ content	Findings
Webb et al. [14]	Healthy subjects	Randomized, open-label crossover	14	Consumption of 500 mL beetroot juice (~ 1395 mg NO_3_)	↓ Both SBP and DBP
Joris et al. [34]	Overweight and obese men	Randomized crossover	20	Consumption of 140 mL beetroot juice (~ 420 mg NO_3_) vs. NO_3_-depleted beetroot juice	Improve postprandial endothelial function
Hobbs et al. [35]	Healthy adults	Randomized, open-label, controlled crossover	23	Daily consumption of 200 g beetroot bread (contains 100 g beetroot ~ 70 mg NO_3_) vs. 200 g white bread (< 0.6 mg NO_3_)	↑ Endothelium-independent vasodilation ↓ DBP
Hobbs et al. [36]	Healthy adults	Randomized, controlled, single-blind, cross-over	32	Consumption of 500 g low-NO_3_ (< 3.1 mg NO_3_) mineral water (as control), 100 g beetroot juice+ 400 g water (~ 142 mg NO_3_), 250 g beetroot juice+ 250 g water (~ 465 mg NO_3_) or 500 g beetroot juice (~ 706 mg NO_3_)	↓ Both SBP and DBP in a dose-dependent manner
Hobbs et al. [36]	Healthy adults	Randomized, controlled, single-blind, cross-over	32	Consumption of 200 g red beetroot- and white beetroot-enriched breads (contains 100 g red- or white-beetroot ~ 112 and 99 mg NO_3_, respectively) vs. white bread (< 3.1 mg NO_3_)	↓ SBP and DBP
Hughes et al. [37]	Healthy young and old adults	Randomized, controlled, double-blind, cross-over	26	Consumption of 500 mL beetroot juice (~ 583 mg NO_3_)	↓ Peripheral and aortic BP in both young and older adults ↓ Aortic wave reflection (assessed by aortic augmentation index) only in young adults
Vanhatalo et al. [30]	Healthy adults	Randomized, controlled, double-blind, cross-over	8	Consumption of 500 mL beetroot juice (~ 322 mg NO_3_) vs. 500 mL low-calorie juice	↓ SBP and DBP
Kapil et al. [38]	Healthy adults	Randomized, double-blind, cross-over	35	Consumption of 250 mL beetroot juice (~ 340 mg NO_3_) vs. 250 mL water	↓ SBP, prevented endothelial dysfunction caused by ischemia reperfusion
Kemmner et al. [39]	patients with chronic kidney disease	Randomized open-label cross-over	17	Consumption of 30 g beetroot powder dispended in 200 mL tap water (~ 300 mg NO_3_) vs. 200 mL tap water	↓ Both SBP and DBP, ↓ arterial BP, ↓ renal resistive index
Ghosh et al. [40]	Hypertensive adults	Randomized open-label crossover	30	Consumption of 250 mL beetroot juice (~ 217 mg NO_3_) vs. 250 mL low NO_3_ water (< 4.3 mg NO_3_)	↓ Both SBP and DBP, ↓pulse wave velocity ↑ Erythrocytic XOR expression and XOR-dependent NO_2_ reductase activity
Velmurugan et al. [33]	patients with hypercholesterolemia	Randomized, double-blind, placebo-controlled	69	Consumption of 250 mL beetroot juice (~ 370 mg NO_3_) vs. NO_3_-depleted beetroot juice	↓ SBP ↑ Flow mediated dilation, ↓pulse wave velocity, ↓ augmentation index
Berry et al. [41]	COPD patients	Randomized, single-blind, crossover	15	Consumption of 140 ml beetroot juice (~ 480 mg NO_3_) vs. NO_3_-depleted beetroot juice (< 1 mg NO_3_)	↓ Resting SBP and DBP
Coles et al. [42]	Healthy adults	Rndomized double-blind,, placebo-controlled, crossover	30	Consumption of 140 ml beetroot juice (~ 465 mg NO_3_) vs. apple juice	↓ SBP in men
Curtis et al. [43]	COPD patients	Randomized double-blind, placebo-controlled, cross-over single dose	21	Consumption of 140 ml beetroot juice (~ 800 mg NO_3_) vs. NO_3_-depleted beetroot juice	↓ Resting DBP

*NO* Nitric Oxide, *DBP* Diastolic Blood Pressure, *SBP* Systolic Blood Pressure, *COPD* Chronic Obstructive Pulmonary Disease

For the first time, Webb et al. performed an open-label cross-over study in healthy volunteers to support the blood pressure lowering properties of a NO_3_ concentrated beetroot juice [14]. This result was confirmed by a meta-analysis of 12 randomized clinical trials by Siervo et al., which highlighted the cardio-protective properties of beetroot juice supplementation in accordance to a significant effect size on systolic blood pressure (SBP) (mean difference = − 4.5, 95% CI = − 6.4, − 2.5) [44]. This study investigated the acute hypotensive properties of beetroot juice, and highlighted the significant association between a daily dose of inorganic NO_3_ (as a biomarker of NO availability, provided as sodium NO_3_ or beetroot juice) and changes in SBP [44].

Additionally, the ingestion of white-beetroot bread (~ 4.5 mg betacyanin/100 g) and red-beetroot bread (~ 27.3 mg betacyanin/100 g), with equivalent doses of NO_3_, is believed to decrease blood pressure to the same extent, affirming a positive linkage among NO_3_ content and the observed blood pressure lowering effect of beetroot [45]. Beetroot juice consumption was also shown to reduce blood pressure, improve endothelial function, and dramatically increase the plasma NO_2_ level and systemic NO production [38]. In healthy subjects, consumption of 500 mL beetroot juice substantially decreased blood pressure in proportion to an increased peak of plasma NO_2_ level [46].

In contrary to the most common conclusion, emphasizing the exclusive role of NO_3_ on the hypotensive effect of beetroot, a recent meta-analysis highlighted the potential NO_3_ independent blood pressure lowering effect and postulated a dose-dependent relationship between inorganic NO_3_ and its hypotensive effect [47]. There are other studies in agreement with this investigation, which have indicated a similar microvascular, vasodilator property following the consumption of NO3- rich beetroot juice, compared to a NO3- depleted placebo, within a period of 24 days [28]; hence, it is suggested that bioactive components other than NO3, may mediate dilatory responses among both beverages [6].

Hypotensive effect of beetroot seems to be highly influenced by physiological and medical status. Beetroot juice administration was found to exert a much stronger effect on blood pressure in hypertensive compared to normotensive subjects, which can be explained by the rate of erythrocyte xanthine oxidase expression (XOR - Erythrocytic Xanthine Oxidoreductase, an enzyme involved in reduction of NO_2_ in active NO) in hypertensive states [40].

As a counterpoint, despite elevations in plasma NO_2_ concentration, no significant decrease of blood pressure has been admitted in diabetic patients supplemented with 250 mL beetroot juice for 2 weeks. The reason for this discrepant result is unclear but may reflect the study methodology, related to concomitant medications or aberrant vascular physiology in diabetic patients [28].

Similarly, the study of Ghosh et al. on 40 hypertensive pregnant women extended our findings on the efficacy of dietary nitrate supplementation in form of 70 mL beetroot juice comparing to control. It was concluded that the ingestion of a single dose of dietary nitrate, does not provide a considerable difference between the two groups at any time points. Although the acute ingestion led to an elevation of diastolic blood pressure (DBP), this measurement returned to the baseline value by the first 24 h and the subsequent first week and therefore, no significant differences in plasma nitrate or nitrite level could be observed. It was however confirmed that a considerable correlation exists among alterations of plasma nitrite conversion and concentration rates, and blood pressure responses, which was consistent with the previous biochemical data and other modalities. With that said, interventions involving dietary nitrate may only appear effective if the individual is capable of undergoing nitrate to nitrite bioconversion or assimilating abilities [40].

The unsustain blood pressure lowering properties, is another substantial topic. A randomized parallel, clinical trial by Jajja et al. among overweight older adults, revealed a progressive decline in SBP measurements following a 3 week beetroot juice supplementation, which returned to the baseline 1 week post-intervention; accordingly, it was concluded that continuous beetroot supplementation might be necessary to sustain beneficial cardiovascular effects [31].

The ultrasound flow-mediated dilatation measurement (FMD) and aortic pulse wave velocity (aPWV) were the primary methods of investigating the potential short and long term effects of beetroot consumption on vascular function. A 6 week supplementation with NO_3_- rich beetroot juice led to a modest improvement of FMD, the aPWV and the augmentation index, and measures of arterial stiffness [45]. Another newly published meta-analysis of clinical studies, reported a significant pooled effect size on FMD following consumption of beetroot juice (standardized mean difference = 0.30, 95% CI = 0.05–0.54), the result of which was similar to impact of pure inorganic NO_3_ (administrated as sodium or potassium NO3) (standardized mean difference = 0.54, 95% CI = 0.21–0.86) [48]. Notably, vascular responsive features to beetroot supplementation can be affected by vascular aging due to a substantial decrease in the NO_3_ to NO_2_ bioconversion capacity; Siervo et al., in a recent meta-analysis, assessed the effect of high NO_3_ beetroot juice on blood pressure variability (24-h ambulatory BP monitoring) and reported a more significant decrease in nocturnal SBP variability in subjects aged < 65 years compared to the older group (≥ 65 y) [49].

In contrary, a 7 day treatment with high- NO_3_ concentrated beetroot juice seemed to have no significant effect on resting or ambulatory blood pressure, aPWV and arterial distensibility among overweight and obese older adults; the plasma concentration of asymmetric dimethylarginine (ADMA), an endogenous inhibitor of NO synthesis and a novel risk marker of cardiovascular disease, were additionally remained unaffected by beetroot supplementation [25].

**Fig. 1:**
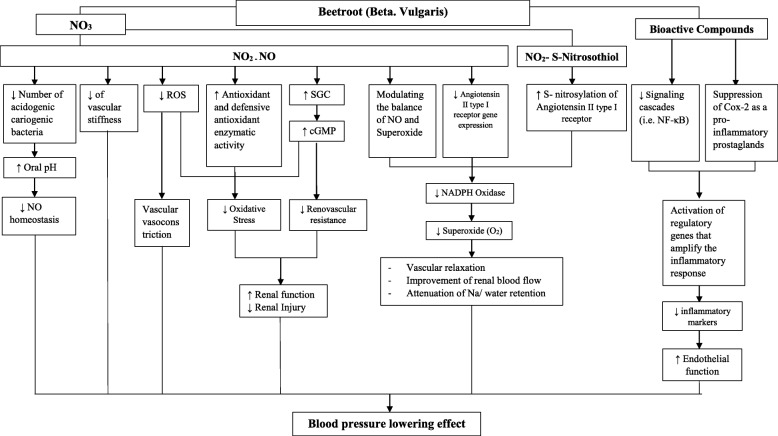
The possible hypotensive mechanisms of beetroot juice in regards to NO_3_ and bioactive compounds; NO_3_ in converted to NO via the NO_3_ - NO_2_- NO pathway. NO decreases the population of acidogenic cariogenic bacteria, increases oral pH, reduces the vascular stiffness and ROS production, and subsequently, improves the endothelial function. Decreased ROS production along with increased activity of antioxidant enzymes, reduce the oxidative stress. NO also activates the SGC- cGMP pathway, which decreases the renovascular resistance and promotes the renal function. NO directly decreases the angiotensin II type I receptor gene expression and produces NO_2_- S- nitrosothiol via the increased S - nitrosylation of angiotensin II type I receptor, which together reduce the NADPH oxidase activity and therefore, improve the renal blood flow and vascular relaxation and change the Na/ water retention. Other bioactive compounds (e.g. polyphenols, betalains, etc.) reduce NF-κB activities, suppress Cox- 2, reduce the inflammatory markers, improved the endothelial function and therefore, reduced the blood pressure. *NO* Nitric Oxide, *ROS* Reactive Oxygen Species, *SGC* Soluble Guanylate Cyclase, *cGMP* Cyclic Guanosine Monophosphate, *NADPH* Nicotinamide Adenine Dinucleotide Phosphate, *NF-κB* Nuclear Factor kappa-light-chain-enhancer of activated B cells

Whether the NO_3_ is responsible for the leading, beneficial effects of beetroot, can be looked at as a controversial topic. The physiological effects of beetroot is suggested to have a direct relationship with its NO_3_ content beyond other bioactive compounds including betacyanins (Fig. 1); mechanisms underlying the hypotensive properties of beetroot is also most likely attributed to an enhancement of NO bioavailability as a result of increased non-enzymatic reduction of NO_3_ into NO_2_ and NO [36, 50]. The pharmacokinetics of NO_3_ are suggested to differ based on the delivery vehicle [51, 52], in which beetroot was used as in the vast majority of clinical studies, investigating the hypotensive effect of NO_3_ [26, 31, 35, 50–52]. The non- significant blood pressure lowering effect of NO_3_- depleted beetroot compared to NO_3_- rich beetroot [32, 41], may imply the key and potential role of NO_3_ versus other beetroot’s nutraceuticals. It is reported that the hypotensive properties of beetroot are proportional to the high turnover of NO_3_/NO_2_ and cyclic guanosine monophosphate (cGMP) plasma level, as the most sensitive indicator of NO bioactivity [14, 38]. Additionally, the blood pressure alterations associated with the consumption of beetroot juice and beetroot-enriched bread were correlated with urinary NO_3_/NO_2_ levels [50]. In a similar instance, there is a considerable trend between the changes in SBP, plasma NO_2_, the reduction of peak and increase of blood pressure and plasma NO_2_ [40].

The NO_3_-mediated hypotensive effect of beetroot is highlighted by the elevation of XOR enzyme expression level and XOR-dependent NO_2_ reductase activity post-ingestion of beetroot juice, and the promised hypotensive effect is disrupted by allopurinol, an XOR inhibitor [40]. The reduction of blood pressure following beetroot consumption is believed to suppress and interrupt the salivary NO_3_ uptake. Various factors such as smoking (increasing salivary thiocyanate) [24], use of antibacterial mouthwash (reduction/ removal of oral NO3- reducing bacteria) [47, 53–55] and inorganic iodide supplementation (salivary NO_3_ uptake reduction) [20] interfere with NO_3_ enter-salivary circulation, a rate-limiting step for dietary NO_3_ metabolism. Evidences suggest this procedure to consequently disturb cardio-protective aspects of dietary NO_3_.

Altogether, based on the current prevailing perception, NO_3_ and its subsequent NO product are mainly responsible for cardio-protective and hypotensive effects of beetroot supplements; while so, additive or synergistic effects of other bioactive compounds such as vitamin C, polyphenols and carotenoids should not be neglected.

### Effects of beetroot on glucose and insulin homeostasis

The potential hypoglycemic effect of beetroot juice across healthy individuals and patients with various disorders have been studied previously, out of which 5 human and 2 animal studies were investigated in this review (Table 4). Significant reduction of blood glucose level and the positive impact on the glycemic and insulin responses were reported (*P*-value= 0.004), among which multiple mechanisms and highlighted role of bioactive compounds (e.g. polyphenols, flavonoids, nitrate etc.) were critical. Due to the contribution of the lipid profile as a complementary factor in the incidence of glycemic abnormalities, this topic was also briefly evaluated in conjunction within this section.

**Table 4:** Effects of beetroot on glucose-insulin homeostasis, lipid profiles and oxidative stress

Author	Study population	Study design	Sample size	Duration (days)	Dose of beetroot and NO_3_	Findings
Shepherd et al. [81]	Healthy young and old adults	Randomized double- blind, placebo controlled, cross- over	31	–	Consumption of 140 mL beetroot juice (~ 738 mg NO_3_) vs. NO_3_-depleted beetroot juice	No effect on plasma glucose, C-peptide, or incretin concentration
Fuchs et al. [82]	Obese, insulin-resistant patients	Randomized double- blind, controlled	16	–	Consumption of 100 mL beetroot juice (~ 300 mg NO_3_) vs. water	No effect on postprandial glucose and insulin response
Wootton-Beard et al. [48]	Healthy adults	Randomized single- blind, placebo controlled, cross- over	16	–	Consumption of 225 mL beetroot juice (~ 990 mg NO_3_) vs. control beverage matched for macronutrient content	↓ Postprandial insulin response in the early phase (0–60 min) ↓ Glucose response in the 0–30 min phase
Gilchrist et al. [28]	T2DM patients	Randomized double- blind, placebo controlled, cross- over	27	14	Daily consumption of 250 mL beetroot juice (~ 500 mg NO_3_) vs. NO_3_-depleted beetroot juice	No effect on insulin resistance
Kerley et al. [27]	Controlled and uncontrolled hypertensive patients	Pilot study	19	14	Daily consumption of 140 ml beetroot juice (~ 800 mg NO_3_)	↓ Serum LDL-C in uncontrolled patients
Asgary et al. [29]	Hypertensive un-treated adults	Randomized crossover pilot study	24	14	Daily consumption of 250 mL beetroot juice vs. 250 g cooked beetroot	No effect on blood glucose ↓ Serum hs-CRP, IL-6 and TNF-α ↑ TAC in beetroot juice group ↓ Non-HDL-C, total cholesterol and LDL-C in beetroot juice group
Singh et al. [80]	Healthy adults	Randomized, cross- over	30	15	Daily consumption of 500 mL beetroot juice	↑ HDL-C, ↓ LDL-C, ↑ Total antioxidant capacity and serum vitamin C levels
Kapil et al. [32]	Hypertensive older adults	Randomized, phase 2, double-blind, placebo-controlled study	68	28	Daily consumption of 250 mL beetroot juice (~ 450 mg NO_3_) vs. NO_3_-depleted beetroot juice	No effect on fasting glucose, glycated haemoglobin, serum creatinine, sodium and potassium, or lipid profile
Velmurugan et al. [33]	patients with hypercholesterolemia	Randomized, double-blind, placebo-controlled	69	42	Daily consumption of 250 mL beetroot juice (~ 370 mg NO_3_) vs. NO_3_-depleted beetroot juice	No effect on oxidized LDL, hs-CRP, and uric acid

*NO* Nitric Oxide, *T2DM* Type 2 Diabetes Mellitus, *hs-CRP* high-sensitivity C-Reactive Protein, *IL-6* Interleukin-6, *TNF-α* Tumor Necrosis Factor-alpha, *HDL-C* High Density Lipoprotein-Cholesterol, *LDL-C* Low Density Lipoprotein- Cholesterol

An observational study was conducted on the phytochemical constituent of 225 mL beetroot juice among 16 healthy adults and related postprandial timing. Three samples of 50 g available carbohydrates, in the form of beetroot juice were administered, with lemon in the first sample, sucrose, fructose, glucose in the second sample (matched control drink) and glucose in the third sample, respectively. This assessment found a positive correlation on both glycemic and insulin responses in the first sample over the two beverages. The glycemic response post- beetroot juice consumption via the first and second drinks was shown to be significantly lower than the third drink. Considerably lower insulin response was elicited between beetroot juice and the control drink that remained non- significant. In this respect, it is suggested that polyphenol- rich beetroot juice might be responsible for the late rise in the early phases of postprandial glucose or insulin responses [48].

Collected data from a recent study on 30 healthy participants outlined further decreasing trend of blood glucose level by 34.5% following longer-term ingestion of a 10% beetroot juice solution [48] within 4 weeks, comparing to the baseline and washout period; whereas such difference was not present within 2 weeks of the interventional phase. With significant assimilation to the hypotensive effect, it can be said that persistent consumption of beetroot juice might be necessary on the maintenance of sustainable impacts of blood glucose and insulin responses [49].

The administration of 270 mL beetroot juice among healthy adults in a randomized cross-over study, suspended the postprandial glycemic response and lowered the sustainability and peak of blood glucose level; therefore in contrast to a sugar-matched control drink, appeared useful [56].

Beals et al. have discussed the augmenting interference of concurrent dietary fiber-rich- beetroot juice and 25 g of glucose (75 g total carbohydrate load) among obese and non-obese individuals with glucose tolerance. Participants were supplemented with 500 mL beetroot solution (17 mmol nitrate and 25 g glucose) at baseline, glucose at time laps of 5, 45, 60 and 90 min, glucose solution and insulin at 10, 20,30 and 120 min. It was eventuated that the inhibition of nitrate reductase activity did not only reduce the desire of metabolic responses to beetroot juice combined with glucose but also promoted insulin resistance (푃 = 0.009) and concealed insulin sensitivity within obese individuals. Unlikely, the co-ingestion of glucose and beetroot juice led to a higher elevation of blood glucose concentration in obese than non-obese adults at 60 and 90 min (푃 = 0.004). It was, therefore, obtained that obese adults with a higher risk of developing insulin resistance, may benefit from nitrate-rich foods [57].

**Fig. 2:**
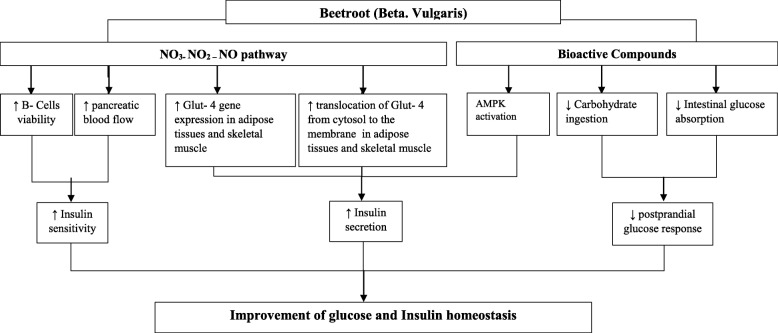
The effect of NO_3_ and other bioactive compounds within beetroot juice on regulation of insulin and glucose homeostasis; NO enhanced the B-cells viability and pancreatic blood flow, which in turn increases the insulin secretion. It also increases the Glut- 4 gene expression and translocation from cytosol to membrane in the adipose tissue and skeletal muscle. This enhancement, along with the activation of AMPK signaling, improves the insulin sensitivity. Reduced carbohydrate digestion and intestinal glucose absorption suppresses the postprandial glucose response as well. The amelioration of insulin sensitivity and insulin secretion, along with the suppression of postprandial glucose response, correspondingly, regulates the insulin and glucose homeostasis. *NO* Nitric Oxide, *AMPK* Adenosine Monophosphate-activated Protein Kinase

Among studies compatible with the blood-glucose-lowering effect of *Beta vulgaris* in this review, multiple mechanisms were suggested to be responsible (Fig. 2). Some papers emphasized the critical role of bioactive compounds [48, 58] including the action of ethanol via an ethanolic extraction of beetroot juice (EEBT) [59]. The nitrite- nitrate pathway, taking place in the oral cavity by the commensal bacteria, was also introduced [57]. Other mechanisms are including the modification of intracellular signal transduction as a major mechanism of reducing blood glucose by foods and hormonal activities, inhibition of α-amylase and α-glucosidase and increase in paraoxonase 1 (PON1) [60]. The increasing trend of serum cortisol level post- beetroot consumption, as a stress hormone and leading factor in the elevation of gluconeogenesis, is coupled with the reduced glucose concentration, as well. This phenomenon can be related to either the Adrenocorticotropic hormone (ACTH) secretion or the mode of action at the adrenal cortex level [49]. Therefore, the intake of beetroot juice decreased the blood glucose level comparing to the control and placebo beverages, the reason of which is postulated to be regarding the polyphenol, betanins and neobetanin, as a betanin degradation product, ethanol content, nitrite- nitrate pathway or the inhibition of hormonal reductase activity.

In addition to the glycemic controlling properties of beetroot juice, some articles highlighted the beneficial effect of this beverage on lipid profile and its parameters (TC, TG, HDL, and LDL), which is directly related to the incidence of T2DM. One randomized, double- blind study in this regard investigated the acute, 2 h post-consumption (2HPP) effect of beetroot juice on plasma glucose and lipid status. Among the related parameters TG was only revealed to be notably higher in the intervention group at pre-treatment level. While so, the 2HPP was shown to be lower than that pre-administration of beetroot juice comparing to the control. The lipid profile parameters decreased at post-treatment level, and all parameters but HDL had significantly lower values than those in the control group. It, however, remained upon discussion whether these implications are to any extent related to α- lipoic acid and antioxidant content of *Beta vulgaris* or other factors involved [58].

An animal study on the blood-glucose-lowering effect of beetroot juice extended our knowledge in an STZ (Streptozotocin) - induced diabetic rat model. In this study, animals have been treated with either 400 mg/ kg p.o. (orally) Ethanolic Extract of Beetroot Juice or 5 mg/ Kg p.o. Glibenclamide. It was implied that the effect of Glibenclamide on TG and cholesterol level was comparable with that in EEBT- treated animals. In fact, administration of EEBT potentially reduced the serum levels of cholesterol and TG in comparison to diabetic control rats, which is hypothesized to be regarding a long exposure (21 days) to EEBT solution [59].

Altogether, these studies raised evidence in favor of blood glucose lowering effect of beetroot and beetroot juice in particular. It can be implied that beetroot juice is able to effectively lessen the impact of insulin resistance in a drug- comparable manner.

### Effects of beetroot on microbiome

Thus far, data and information regarding the impact of *Beta vulgaris* on gut microbiome and salivary microflora is limited, yet the association with metabolic dysfunction cannot be neglected. Within this context it was primarily indicated that dietary NO_3_ supplementation could alter the salivary microbiome, an outcome that has been perused through investigation of 6 human and 7 animal studies in this review.

Vanhatalo et al. have performed one of the leading human studies on the connection between the nitrate-responsive oral microbiome and nitric oxide (NO) homeostasis, which have revealed a positive relationship between nitrate- nitrite pathway on microbiome action and salivary flow rate. Nevertheless, it is still in doubt, how the abundance of known NO_3_- reducing bacteria such as Fusobacterium nucleatum, Prevotella melaninogenica and Leptorichia buccalis affects nitrate response. NO biomarkers, including the blood pressure and arterial stiffness, have been used to express the results. The NO_3_ and NO_2_ values were seen to be significantly higher in the beetroot juice supplemented group than the control. The results have also explained no considerable SBP and DBP modulations comparing to the baseline, notwithstanding a non- significant difference to be present among older subjects, due to the higher NO_2_ concentration in that group following beetroot juice consumption. The high baseline abundance of Fusobacterium nucleatum subsp. vincentii and nucleatum among other oral bacteria reduced blood pressure as a physiological response to beetroot juice supplementation as the phyla that possess a non-significant lower availability following beetroot juice consumption comparing to placebo [61]. Based on this article, the chronic ingestion of inorganic NO_3_ not only increases a proportion of the oral microbiome including Bacteroidetes, Firmicutes and Fusobacteria, but also serves to change the relative abundance of a few, but not all, NO_3_- reducers. This alteration is positive in Neisseria and Rothia reducers, due to the high NO bioavailability, as a probable cardiovascular health promoter, and negative among Prevotella and Veillonella. Authors concluded that dietary NO_3_ supplementation could alter the salivary microbiome in young and old normotensive individuals [61].

The consumption of nitrate-rich *Beta vulgaris* also increase the consequent rate of NO bioconversion and mean pH (from 7.0 to 7.5). Assuming bioconversion to occur in the mouth [62], it is suggestive that this process may play a critical role in host defense [63, 64], lower prevalence of metabolic dysfunction and caries in the oral cavity through acidification- preventing properties of human saliva and therefore, shift the composition of the microbiome [62]. It is well established that the administration of NO_3_ supplementation as beetroot juice enhances cardio-protective and cardio-enhancing properties [65–67]. In one study, 46 healthy participants were treated with 100 mL of beetroot juice or placebo, (each corresponding to 400 mg and 2 mg NO_3)_. Collected data indicated that the salivary nitrate concentration among beetroot juice and placebo consuming groups both had elevated, yet the range in beetroot juice consumers reached more significant. Consequently, it was highlighted that the baseline value reached the maximum amount within day 8 of beetroot juice and day 15 of placebo consumption [62].

Since the NO bioconversion is of high importance in the metabolic function, the second assessment was performed on the total NO bioavailability and bioconversions in the mouth, as a commensal microflora- dependent procedure. Based on the measurements, the ultimate levels were detected within the first 2 h subsequent to each drink, comparing to the basal mean levels of salivary NO. NO concentration was found to decline among the beetroot juice consuming group and back to basal levels straight after the juice consumption period, suggesting consistent ingestion for the determination of antimicrobial effect and other biological functions of NO to be required [62].

The fermentation of beetroot juice has recently sparked interest as an evolving strategy that is being investigated across several human and animal subjects. One of such efforts is a study on Lactic acid bacteria fermentation, where three phylus of *Lactobacillus plantarum*, Lactobacillus rhamnnosus, and Lactobacillus delbrueckii sb. were cultivated on pasteurized beetroot juice. The comparison of fresh and probiotic beetroot juice, presented a slight increase in the protein values from 3.74 to 3.77%, the acidity of the samples from 0.49 to 0.78% and total antioxidant activity and capacity [68].

This conclusion was in agreement with a recent study among animal models, investigating the administration of lacto-fermented beetroot juice (FBJ) alone or along with M-nitroso-N-methyl urea (MNU- as a harmful factor). The results have shown Bifidobacterium to be the most stable microorganism that almost equally colonized the gut epithelium. It also indicated that the mutagen MNU is incapable of affecting microorganism adherence to the gut epithelium. MNU led to various outcomes depending on the bacterial phylus and the type of intervention [69].

Fresh and lacto-fermented beetroot juices are distinguished by the high anti-carcinogenic and anti-mutagenic potentials [70–72]. Betacyanin components of FBJ, betanidin and betanin overwhelms that in the fresh juice, which is consist of betanin as a dominating compound, instead of betanidin [73]. This study displayed an increase in the antioxidant capacity of blood serum in groups administered with FBJ [69].

The same author has studied the effect of probiotic Lactobacillus casei 0920 and Lactobacillus brevis 0944 fermented beetroot juice (beetroot juice as a lactic acid bacteria carrier). Accordingly, it was concluded that the consumption of fermented juice containing live lactic acid bacteria could positively change the count of intestinal microflora, its metabolic activity, and enzymes involved in the process of carcinogenesis including β-glucosidase, β-glucuronidase, and β-galactosidase. In other words, the daily administration of fermented beetroot juice reduced the enzymatic activity to 75.4, 53.6, and 59.5 U/g, respectively. The activity of β – glucuronidase was also decreased subsequent to the administration of 3.0 and 6 mL of the fermented beetroot juice per day (by 26 and 28%, respectively) [74].

Therefore, it was elucidated that chronic and regular ingestion of fermented beetroot juice may lead to the sustaining intestinal microbial ecosystem and modifying the metabolic activity to reduce the risk of food intolerance related diseases [74].

### Effects of beetroot on kidney function

Despite the positive hypertensive and hyperglycemic effect of beetroot juice, a limited number of studies have acknowledged the reno-protective properties associations with specific renal parameters. To address the key areas of this topic in our comprehensive review, 6 selective literatures, 3 human and 3 animal studies, were summarized and reported. Beetroot juice consumption and its ultimate outcomes in this section, appeared more beneficially among animal models.

In this regard, one and the main human study on stages 2 to 5 of Chronic Kidney Disease patients (CKD II-IV any degree of decrease in the renal function) by Kemmner et al., suggested the administration of nitrate donor beetroot juice to a nitrate load of 300 mg across 9 patients to increase (NO) concentration and elevate the renal resistive index (RRI) as prognostic markers for cardiovascular mortality [39]. This outcome was more vivid among CKD patients that faced a reduction of renal function and elevation of arterial stiffness, with Glomerular Filtration Rate (GFR) values below normal of 90 mL/min/m^2^. This decreased value was primarily caused by hypertensive or diabetic nephropathy, both as causal factors or the subsequent results of the failure. Comparing to the control, the serum creatinine, GFR and serum potassium level did not alter significantly following beetroot juice ingestion, which in case of potassium, also remained about persistent comparing to placebo [39].

With that said, an animal intervention has investigated the beneficial contribution of either beetroot juice or nutraceutical beetroot juice in the treatment of Gentaciamin-nephrotoxicity- induced rats. It was suggested that beetroot juice with prophylactic perspectives actively supported the renal system to overcome the adverse effects of Gentamicin (GM)'s primary and secondary reactive metabolites, resulting from the toxicant-induced damage. Therefore, consumption of beetroot- based beverages depicted positive implications by increasing the level of Superoxide Dismutase (as a primary antioxidant enzyme), and Catalase (involved in a detoxification procedure), while decreasing NO (with a controversial role in renal system), and oxidative stress, all as renal tissue-specific markers. Similarly, urea and creatinine content have lowered, while the protein profile of beetroot- based beverage accelerated, due to the action of bioactive compounds like betacyanins and betaxanthin [75].

Several protective strategies have been introduced to hold effective reno-protective implications. The blood pressure lowering effect via the action of Guanylyl Cyclases and cGMP, and subsequent nitrate- nitrite pathway of the facultative bacteria [76], a nitrate- mediated reduction of renal oxidative stress via decreasing the NADH oxidase activity and angiotensin II receptor (signals that attenuate angiotensin II-mediated renal arteriolar contraction) [77, 78] are of all conclusions drawn to explain the mechanism of action.

An animal study investigated the advantage of beta- vulgaris ethanol extract (BVEE) with potent antioxidant, anti-inflammatory and reno-protective properties in the treatment of GM- induced nephrotoxicity, modulation of renal dysfunction, oxidative stress, inflammation and amelioration of histological damage in rats. The administration of BVEE and GM- treatment subsequently, substantiated a significant suppressing effect on the elevation of urea, uric acid, total protein and creatinine in a dose-dependent manner [79].

BVEE beverage (250 and 500 mg/kg, p.o) was also suggested to be practical on kidney lipid peroxidation factors. The activity level of catalase, as an important antioxidant enzymes, was reduced by 27.97% following GM treatment, and notably increased by 83.92 and 92.62%, respectively, following the administration of 250 and 500 mg/kg BVEE. Similar trend was present for NP- SH content (non- protein sulfhydryl- for the measurement of renal non- protein sulfhydryl); a reduction of 37.94% following 85 mg/kg GM treatment was present, comparing to 71 and 81.71% increase in 250 and 500 mg/kg BVEE, respectively. The total protein content of GM treated animals was decreased by 71.46% in comparison with a dose dependent increase in pretreated groups of 250 mg/kg and 500 mg/kg BVEE by 37.35 and 43.74%, respectively [79].

The data and findings here confirmed the ameliorating effect of *Beta vulgaris* as a beneficial additive treatment on kidney’s functional parameters, reducing the progressive rate of renal disease, and subsequently mortality in high risk groups including hypertensive CKD and diabetic nephropathy patients. It however remains to be investigated whether the decreasing effect over RRI values and blood pressure is ascribed to supplementation with the vasodilator- dietary nitrate or potent antioxidant, anti-apoptosis and anti-inflammatory properties possess by betacyanin components including betanin and betanin. It is upon discussion that BVEE treatment improves the extent of structural damage and decreases inflammatory infiltration in renal tubules through the reduction of oxidative stress, inflammation, and apoptosis in the kidney.

### Potential drug interaction and adverse effects

There is limited evidence in regards to the adverse effects and tolerance issues of beetroot juice and its components. Exclusive number of studies reported major negative implications associated with the consisting bioactive compounds, out of which, 5 studies were assessed in our review.

Beeturia, urea discoloration or excretion of red/ pink urine following beetroot ingestion occurs due to the presence of un-metabolized betalain pigments in the urine and has been reported in 10–14% of Shepherd’s study population [17]. It is a strong, though benign effect that had been stated by most of the participants of previous studies as well. In other words, short term and long term treatment with beetroot juice were well tolerated by the subjects. This is a confirmation on a safe administrating strategy of beetroot through acute and chronic phases [18].

It is partly evident that a 5 days administration of betalain- rich beetroot juice (25 and 100 mg∙kg∙bm − 1), markedly inhibited NF-κB DNA-binding activity in renal damage- induced rats and significantly suppressed Cyclooxygenase 2 (Cox- 2) expression in vitro by nearly 97%. This revealed a more significant anti-inflammatory effect than many rival synthetic drugs including Ibuprofen, Vioxx and Celebrex [4]. It is also proven that a 28 days administration of beetroot juice (250 mg or 500 mg∙kg∙bm − 1), inhibits NF-κB DNA binding activity across nephrotoxic rats in a dose-dependent manner. Therefore, an alternative here to synthetically manufactured medications, including Non- steroidal anti-inflammatory drug (NSAIDs), is to shift towards natural resources and additional treatments [4].

The potential interaction of NO_3_- rich beetroot juice with phosphodiesterase-5 inhibitors and consequent severe hypotension, is another consideration. Beneficial properties of beetroot can be profoundly affected by medications imposing undesirable interaction with metabolism and the ultimate metabolic pathways of NO_3_/ NO_2_; as such, the hypotensive effect of orally ingested NO_2_ is proposed to be abolished by esomeprazole, a proton pump inhibitor [18].

Beetroot itself takes crucial part in drug metabolism and pharmacological treatment; the carotenoid content is said to be involved in Xenobiotic function and metabolism. The interference of Betalain with a broad spectrum of anti- inflammations properties and the pro-inflammatory signaling agent especially the Nuclear Factor-Kappa B (NF-κB) cascade is taken into account as an alternative of therapeutic medications with fewer adverse effects [4].

Cytochrome P450 (CYP450) is known to be an active transporter in drug metabolism. This hemeprotein, with potent vasoconstriction properties, and its metabolite 20-Hydroxyeicosatetraenoic acid (20-HETE) are critical in regulation of renal, pulmonary and cardiac function [80]. To the best of our knowledge, there are no studies in regards to the direct effect of beetroot and its byproducts on CYP450, however, NO, as an important beetroot component, was revealed to mediate the inflammatory- induced down- regulation of CYP450 and therefore, inhibit the conversion of CYP450 to 20-HETE. Also, active components including polyphenols, flavonoids and anthocyanins possess a similar inhibitory response in a comparable manner to other CYP450 inhibitors. Therefore, the food- drug and drug- food interactions along with the dose and duration of drug intake come back into focus.

Subsequently, it is important to ascertain the probable interactions between beetroot juice and various supplements of proven ergogenic effects such as caffeine, creatinine, β-alanine, and sodium bicarbonate, and assure the possible beneficial impacts [7].

With accordance to the high oxalic acid constitution of beetroot, comparing to other vegetables and fruits (400–600 mg/100 g fresh weight) [1], natural beetroot- based supplements, are not presently anticipating major negative health outcomes related to beetroot juice bioactive components. Therefore, it is unlikely to be a risk to human health in the short term.

Considering the public interest to ergogenic and cardio-protective effects of beetroot supplementation, future clinical studies are required to evaluate the long-term efficacy and safety of beetroot dietary intervention in health and disease states.

## Conclusion

Available data supported the health-promotional properties of beetroot and its byproducts, as a potential therapeutic treatments for various metabolic disorders including hypertension, diabetes, insulin resistance and kidney dysfunction. In human studies to date, beetroot supplementation has been reported to reduce systolic and diastolic blood pressure, inhibit platelet aggregation, improve vascular and endothelial function, reduce blood glucose and improve insulin homeostasis, and possess reno-protective properties. Beetroot contains high concentration of phytochemicals and essential nutrients and is abundant in inorganic NO_3_. Bioactive compounds are believed to play crucial roles within the mechanistic pathways and be responsible for the promising clinical effects.

## Data Availability

Not Applicable.

## Abbreviations

aPWV: Aortic Pulse Wave Velocity
BMI: Body Mass Index
BP: Blood Pressure
CKD: Chronic Kidney Disease
CYP450: Cytochrome P450
DBP: Diastolic Blood Pressure
FBS: Fasting Blood Sugar
HDL: High-Density Lipoprotein
HETE-20: 20-Hydroxyeicosatetraenoic acid
LDL: Low-Density Lipoprotein
NO: Nitric oxide
NO_2_: Nitrite
NO_3_: Nitrate
SBP: Systolic Blood Pressure
T2DM: Type 2 Diabetes Mellitus
TC: Total Cholesterol
TG: Triglyceride
